# Exposome-wide association study of environmental chemical exposures and epigenetic aging in the national health and nutrition examination survey

**DOI:** 10.18632/aging.206201

**Published:** 2025-02-11

**Authors:** Dennis Khodasevich, Nicole Gladish, Saher Daredia, Anne K. Bozack, Hanyang Shen, Jamaji C. Nwanaji-Enwerem, Belinda L. Needham, David H. Rehkopf, Andres Cardenas

**Affiliations:** 1 Department of Epidemiology and Population Health, Stanford University, Palo Alto, CA 94305, USA; 2 Division of Epidemiology, Berkeley Public Health, University of California, Berkeley, Berkeley, CA 94720, USA; 3 Department of Emergency Medicine, Perelman School of Medicine, University of Pennsylvania, Philadelphia, PA 19104, USA; 4 Department of Epidemiology, University of Michigan, Ann Arbor, MI 48109, USA; 5 Department of Health Policy, Stanford University, Palo Alto, CA 94305, USA; 6 Department of Medicine (Primary Care and Population Health), Stanford University, Palo Alto, CA 94305, USA; 7 Department of Pediatrics, Stanford University, Palo Alto, CA 94305, USA; 8 Department of Sociology, Stanford University, Palo Alto, CA 94305, USA; 9 Center of Excellence in Environmental Toxicology, Perelman School of Medicine, University of Pennsylvania, Philadelphia, PA 19104, USA

**Keywords:** epigenetic aging, environmental exposures, exposome, epigenetics, aging

## Abstract

Epigenetic clocks can serve as pivotal biomarkers linking environmental exposures with biological aging. However, research on the influence of environmental exposures on epigenetic aging has largely been limited to a small number of chemicals and specific populations. We harnessed data from the National Health and Nutrition Examination Survey 1999-2000 and 2001-2002 cycles to examine exposome-wide associations between environmental exposures and epigenetic aging. A total of 8 epigenetic aging biomarkers were obtained from whole blood in 2,346 participants ranging from 50-84 years of age. A total of 64 environmental exposures including phthalates, metals, pesticides, dioxins, and polychlorinated biphenyls (PCBs) were measured in blood and urine. Associations between log_2_-transformed/standardized exposure measures and epigenetic age acceleration (EAA) were assessed using survey-weighted generalized linear regression. A 1 standard deviation (SD) increase in log_2_ serum cadmium levels was associated with higher GrimAge acceleration (beta = 1.23 years, p = 3.63e-06), higher GrimAge2 acceleration (beta = 1.27 years, p = 1.62e-05), and higher DunedinPoAm (beta = 0.02, p = 2.34e-05). A 1 SD increase in log_2_ serum cotinine levels was associated with higher GrimAge2 acceleration (beta = 1.40 years, p = 6.53e-04) and higher DunedinPoAm (beta = 0.03, p = 6.31e-04). Associations between cadmium and EAA across several clocks persisted in sensitivity models adjusted for serum cotinine levels, and other associations involving lead, dioxins, and PCBs were identified. Several environmental exposures are associated with epigenetic aging in a nationally representative US adult population, with particularly strong associations related to cadmium and cotinine across several epigenetic clocks.

## INTRODUCTION

Epigenetics is the study of modifications that influence gene expression without changes to the underlying DNA sequence. Epigenetic biomarkers, including DNA methylation (DNAm), can provide insights to pathways linking environmental exposures to health outcomes [1]. In addition to imparting information about the regulation of gene expression, DNAm changes consistently with age and DNAm data can impart meaningful information on the role of various factors in shaping the biological aging process through the use of epigenetic aging biomarkers, also referred to as epigenetic clocks. Epigenetic clocks include several predictors of chronological age, health-related biomarkers, and mortality based on observed DNA methylation levels of specific CpG sites throughout the genome [2]. First generation epigenetic clocks were trained to predict chronological age and include the Horvath panTissue, Skin&Blood, and Hannum clocks [3–5]. In contrast, second and third epigenetic clocks were designed to predict different aspects of the biological aging process, such as mortality, frailty, telomere length, and the pace of aging, and include GrimAge (version 1 and version 2), PhenoAge, DNAmTL, and DunedinPoAm [6–10]. Deviations between an individual’s chronological age and the predictions from a given epigenetic clock, commonly referred to as epigenetic age acceleration (EAA), have been associated with numerous health factors including longevity, cardiovascular disease, and cancer [11]. Epigenetic clocks may reflect the impact of environmental exposures on age-related pathways, and research has revealed associations between several environmental exposures, including air pollution and per- and polyfluoroalkyl substances, and altered epigenetic aging [12].

Most research on the environmental determinants of epigenetic aging has focused on hypothesis-driven research on well-studied chemicals. Although this approach has provided key insights into the role environmental chemicals have in shaping biological aging processes, this approach may impede the discovery of novel associations. An exposome-wide association study (ExWAS) design provides a framework for the systematic examination of associations between a collection of environmental exposures and a phenotype of interest, enabling the discovery of novel associations [13]. However, ExWAS designs have been sparingly applied in epigenetic aging research. One previous ExWAS performed in a sample of 1,173 seven-year-old children from the HELIX project cohort demonstrated the utility of this approach, where in addition to finding expected associations, such as between maternal smoking and EAA, also uncovered several novel associations related to particulate matter, pesticides, and polychlorinated biphenyls (PCBs) [14]. Relatedly, this ExWAS approach has been used to identify several associations between environmental exposures including cadmium and PCBs with telomere length, another biomarker of biological aging, within the National Health and Nutrition Examination Survey (NHANES) [15].

Leveraging the success of previous studies, we sought to perform an ExWAS to measure cross-sectional associations between environmental chemical exposures and EAA among adult NHANES participants during the 1999-2000 and 2001-2002 survey cycles. NHANES is a program designed to assess the health status of a nationally representative sample of adults and children in the United States (US) [16]. NHANES provides a valuable resource with cross-sectional demographic, questionnaire, and high-quality laboratory measures in a large number of US participants, including a vast array of environmental exposures making this an optimal cohort to validate previously identified associations and discover novel associations with epigenetic clocks.

## RESULTS

### Study sample characteristics

The overall distribution of relevant demographic variables in the NHANES study sample, prior to the application of sample weights, are presented in Table 1. NHANES participants ranged from 50 to 84 years of age, with a mean age of 65.1 years. There was a relatively even distribution of males (51.2%) and females (48.8%) in the study sample. The unweighted sample was predominantly Non-Hispanic White (39.3%), with the remaining population makeup being 29.0% Mexican American, 21.8% Non-Hispanic Black, 6.4% Other Hispanic, and 3.5% other/multi-racial. White-collar (high skill) was the most common occupation category (41.7%), followed by blue-collar (semi-routine) (23.5%), white collar (semi-routine) (17.9%), blue collar (high skill) (14.1%), and never worked (2.7%).

**Table 1:** Sample demographics of adults from NHANES 1999-2000 and 2001-2002 with available DNA methylation data.

**Demographic Variable**	**Overall**
**N**	2,346
**Age (Years)**	65.10 (9.29)
**BMI (kg/m2)**	28.80 (5.83)
**Sex**	
Male	1,202 (51.2%)
Female	1,144 (48.8%)
**Race/Ethnicity Category**	
Mexican American	681 (29.0%)
Other Hispanic	151 ( 6.4%)
Non-Hispanic White	922 (39.3%)
Non-Hispanic Black	511 (21.8%)
Other Race - Including Multi-Racial	81 ( 3.5%)
**Poverty to Income Ratio**	2.61 (1.60)
**Education**	
High School Diploma (including GED)	487 (20.8%)
Less Than High School	1,063 (45.3%)
More Than High School	794 (33.9%)
**Occupation**	
Blue-collar (high skill)	312 (14.1%)
Blue-collar (semi-routine)	520 (23.5%)
White-collar (high skill)	921 (41.7%)
White-collar (semi-routine)	396 (17.9%)
Never worked	60 ( 2.7%)
**Smoking Category**	
Current	374 (16.0%)
Former	903 (38.6%)
Never	1,063 (45.4%)

Mean (SD) presented for continuous variables and counts (%) given for categorical variables. Unweighted sample sizes and distributions are presented.

**Figure 1:**
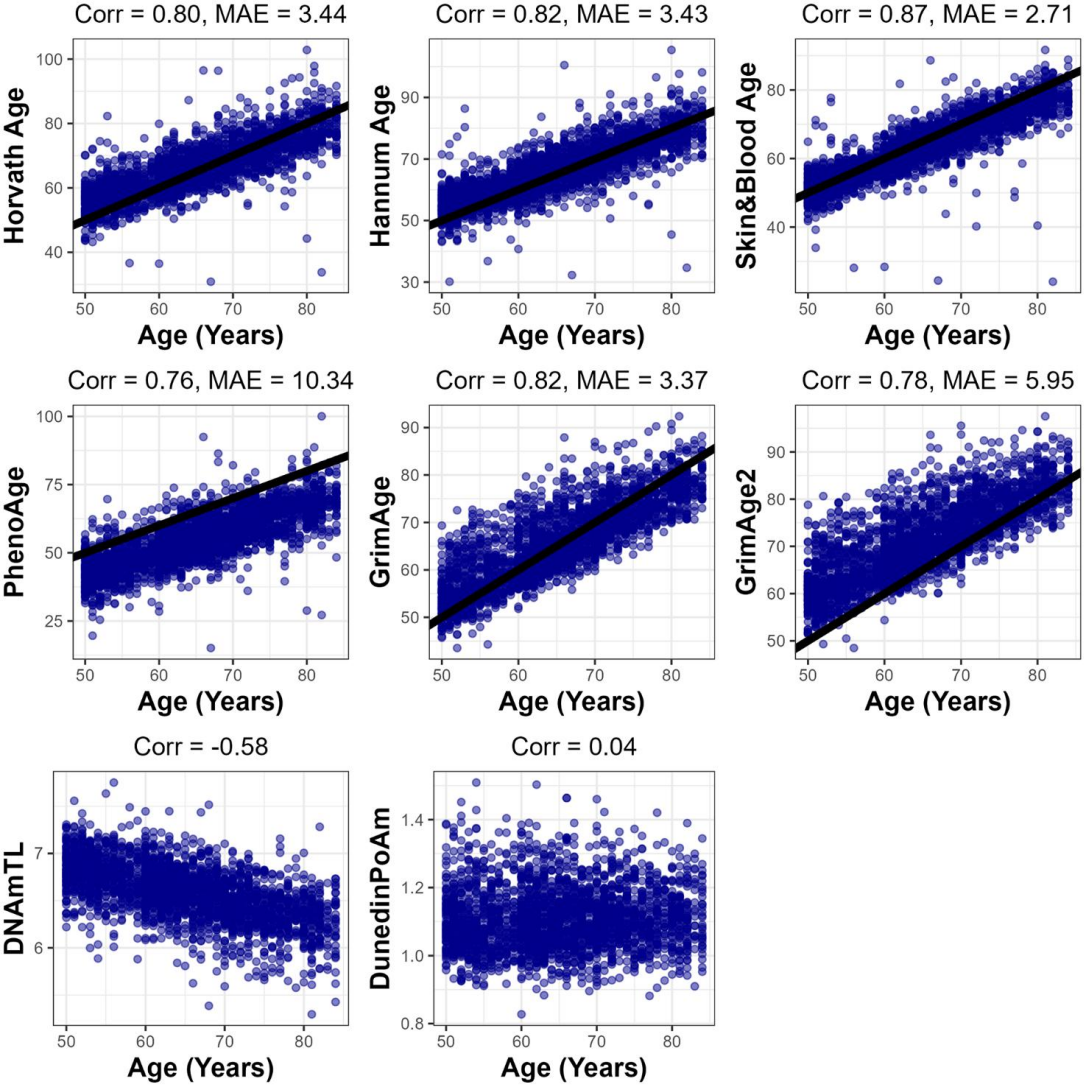
**Fit between each epigenetic clock predictions and chronological age.** Pearson correlation (Corr.) and median absolute error (MAE) presented for each clock which has years for units. 1-to-1 line shown in black for main clocks.

Each of the epigenetic clocks, except for DunedinPoAm and DNAmTL, exhibited a strong positive correlation with chronological age, ranging from *r* = 0.76 for PhenoAge to *r* = 0.87 for Skin&Blood. (Figure 1) Median absolute error between epigenetic age and chronological age ranged from 2.71 years with Skin&Blood to 10.34 years with PhenoAge. Modestly lower correlation and higher MAE with chronological age in the NHANES sample may be explained by missing probes for some clocks on the EPICv1 array or the diversity of the NHANES population. The DNAm-based predictor of telomere length DNAmTL exhibited a negative correlation of *r* = -0.58 with chronological age, indicating the shortening of telomere length with increasing age as expected, and DunedinPoAm was uncorrelated with chronological age (*r* = 0.04). Most EAA measures were moderately correlated with each other, with the highest observed correlation between GrimAge acceleration and GrimAge2 acceleration (*r* = 0.97), and the lowest magnitude correlation between DunedinPoAm and Skin&Blood acceleration (*r* = 0.09) (Supplementary Figure 1). As expected, DNAmTL acceleration was negatively correlated with each of the EAA measures.

Our systematic analysis included lead, cadmium, & cotinine measured in blood, 24 dioxins, furans, & coplanar PCBs measured in serum, 9 metals measured in urine, 6 pesticides measured in urine, 11 phthalates, phytoestrogens, & polyaromatic hydrocarbons (PAHs) measured in urine, and 11 volatile organic compounds (VOCs) measured in blood. A visualization of the Pearson correlation coefficients between all environmental exposures is presented in Supplementary Figure 2. Positive correlations were observed between several of the measured PCBs; however, we note that incomplete overlap in participants selected for each environmental exposure limits the ability to systematically evaluate correlation among all of the surveyed exposures. Sample sizes for complete case analysis for each exposure ranged from 1,893 with blood lead and cadmium exposure to 96 with methyl t-butyl ether exposure, with the median sample size being 577.5. Sample sizes for all exposures, detection frequencies, and survey-weighted exposure distributions are summarized in Supplementary Table 1. We note that sample sizes of VOCs were too low to allow for the fitting of most models examined here, except for the covariate-imputed non-cell adjusted models, but we include VOCs in the number of exposures for the multiple testing p-value adjustment for each model series to maintain stringent control for the number of models selected *a priori*.

### Primary analysis

**Figure 2:**
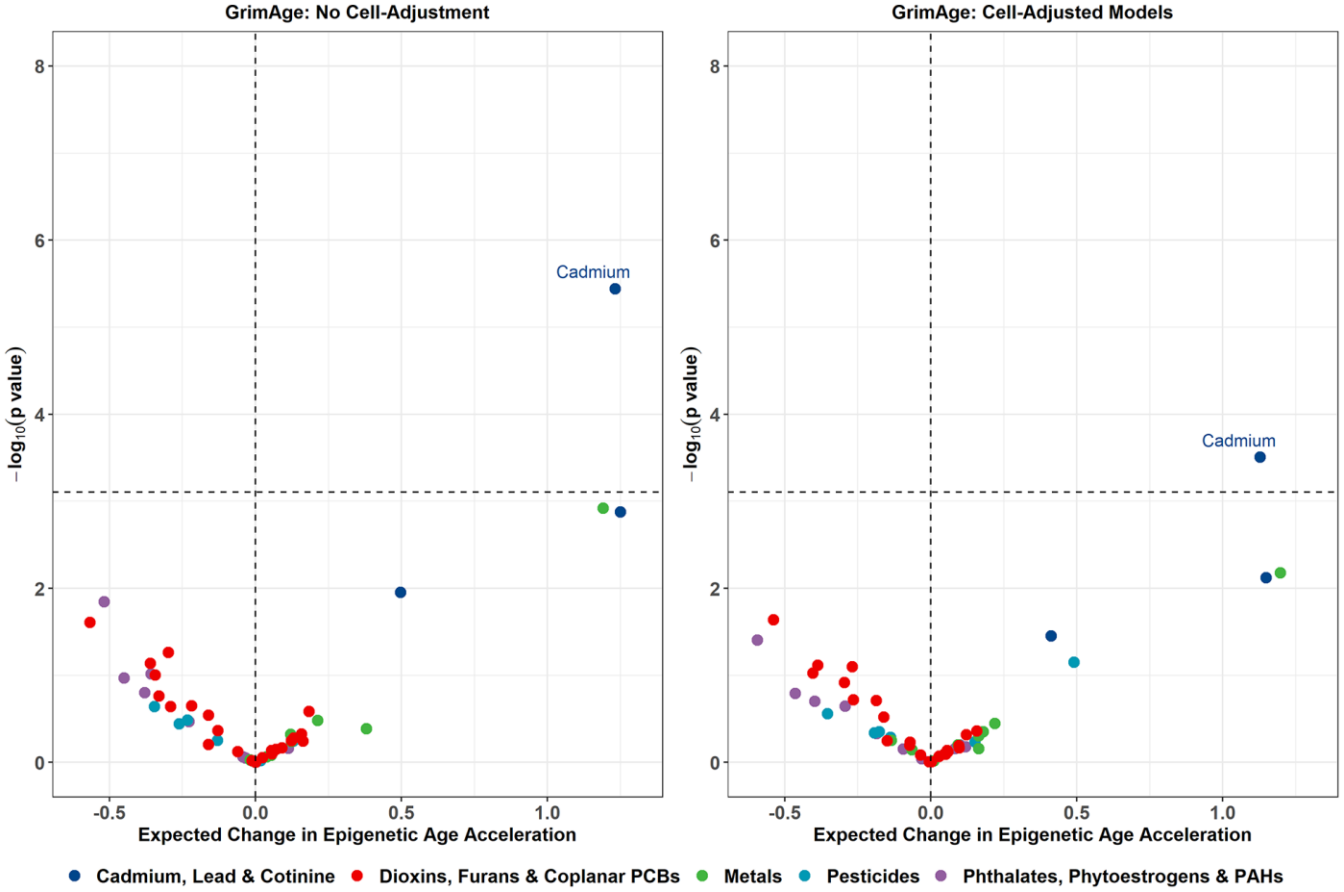
**Volcano plots displaying the expected change in GrimAge acceleration for a 1 SD increase in log_2_-transformed exposure on the X-axis, and -log_10_ p-values on the Y-axis from the primary models.** Color corresponds to the broad category of exposure. Volcano plots for remaining epigenetic clocks are presented in Supplementary Figure 3.

In the primary models without cell-type adjustment, strong associations of serum cadmium and serum cotinine levels with EAA measures were observed for several epigenetic clocks (Table 2). A 1-SD increase in log_2_ cadmium exposure was associated with higher GrimAge acceleration (beta = 1.23 years, p = 3.63e-06), higher GrimAge2 acceleration (beta = 1.27 years, p = 1.62e-05), and higher DunedinPoAm (beta = 0.02, p = 2.34e-05). A 1-SD increase in log_2_ cotinine exposure was associated with higher GrimAge2 acceleration (beta = 1.40 years, p = 6.53e-04) and higher DunedinPoAm (beta = 0.03, p = 6.31e-04). These associations were attenuated after adjusting for estimated cell proportions, with only the association between cadmium exposure and GrimAge (beta = 1.13 years, p = 3.12e-04) and between cadmium and GrimAge2 (beta = 1.12 years, p = 6.13e-04) remaining significant after multiple testing adjustment. Volcano plots visualizing the effect estimates and unadjusted p-values for all exposures from the GrimAge models are shown in Figure 2, with the volcano plots for the remaining clocks displayed in Supplementary Figure 3.

**Table 2:** Primary model summary table.

**Exposure**	**Clock**	**Estimate**	**95% CI**	**P-Value (Raw)**	**Adj. P-Value (Bonferroni)**
*No cell-adjustment*					
Cadmium	GrimAge	1.23	( 0.91, 1.55)	3.63E-06	2.32E-04
Cadmium	GrimAge2	1.27	( 0.88, 1.65)	1.62E-05	1.04E-03
Cadmium	DunedinPoAm	0.02	( 0.01, 0.03)	2.34E-05	1.50E-03
Cotinine	DunedinPoAm	0.03	( 0.01, 0.04)	6.31E-04	0.04
Cotinine	GrimAge2	1.40	( 0.74, 2.05)	6.53E-04	0.04
					
*Cell-Adjusted*					
Cadmium	GrimAge	1.13	( 0.80, 1.46)	3.12E-04	0.02
Cadmium	GrimAge2	1.12	( 0.75, 1.50)	6.13E-04	0.04

Effect estimates, 95% Confidence Intervals (CI), raw p-values, and Bonferroni-adjusted p-values for each exposure found to be significant after multiple testing adjustment from the no cell-adjustment and cell-adjusted models.

Given that tobacco is a major source of cadmium exposure, we further explored the role of smoking status by examining associations between blood cadmium exposure and epigenetic clocks stratified by smoking status for all observations found to be significant in the no cell-adjustment models. Cadmium exposure was strongly associated with higher GrimAge acceleration among former smokers (beta = 2.00 years, p = 2.77e-06) and current smokers (beta = 1.28 years, p = 1.55e-03), while the association among never smokers (beta = 0.07 years, p = 0.75) was positive but nonsignificant. Similarly, the association between cadmium exposure and GrimAge2 acceleration was only significant among former smokers (beta = 1.95 years, p = 3.11e-05) and current smokers (beta = 1.32 years, p = 3.27e-03). The association between cadmium exposure and DunedinPoAm was only significant among former smokers (beta = 0.03, p = 6.49e-05), while the associations among current smokers (beta = 0.01 years, p = 0.13) and never smokers (beta = 0.01, p = 0.20) were positive but nonsignificant.

### Sensitivity analyses

To better determine the extent to which associations might be driven by current smoking activity, we performed a sensitivity analysis rerunning the primary models while adjusting for serum cotinine levels in place of self-reported smoking behavior. Cadmium exposure retained its associations with higher GrimAge acceleration (beta = 1.94 years, p = 1.69e-07), higher GrimAge2 acceleration (beta = 1.97 years, p = 6.80e-07), higher DunedinPoAm (beta = 0.03, p = 8.13e-07), and exhibited an association with shorter DNAmTL (beta = -0.04, p = 4.35e-04) in the non-cell-adjusted models (Table 3).

**Table 3:** Sensitivity model summary table.

**Exposure**	**Clock**	**Estimate**	**95% CI**	**P-Value (Raw)**	**Adj. P-Value (Bonferroni)**
*No Cell-Adjustment*					
Cadmium	GrimAge	1.94	( 1.54, 2.33)	1.69E-07	1.07E-05
Cadmium	GrimAge2	1.97	( 1.52, 2.43)	6.80E-07	4.28E-05
Cadmium	DunedinPoAm	0.03	( 0.02, 0.04)	8.13E-07	5.12E-05
Cadmium, urine	GrimAge	2.14	( 1.54, 2.75)	8.34E-06	5.26E-04
Cadmium, urine	GrimAge2	2.32	( 1.58, 3.07)	2.64E-05	1.66E-03
Lead	GrimAge	0.73	( 0.46, 1.01)	8.80E-05	5.54E-03
HpCDD	GrimAge	-1.21	(-1.70, -0.71)	2.01E-04	0.01
Cadmium, urine	DunedinPoAm	0.03	( 0.02, 0.04)	2.35E-04	0.01
Lead	GrimAge2	0.67	( 0.37, 0.98)	3.90E-04	0.02
Cadmium	DNAmTL	-0.04	(-0.05, -0.02)	4.35E-04	0.03
PCB118	GrimAge	-1.06	(-1.55, -0.57)	5.22E-04	0.03
HpCDD	GrimAge2	-1.18	(-1.74, -0.62)	6.07E-04	0.04
PCB118	GrimAge2	-1.14	(-1.69, -0.58)	7.50E-04	0.05
					
*Cell-Adjusted*					
Cadmium	GrimAge	1.86	( 1.45, 2.27)	3.25E-05	2.05E-03
Cadmium	GrimAge2	1.88	( 1.41, 2.34)	6.38E-05	4.02E-03
Cadmium	DunedinPoAm	0.03	( 0.02, 0.03)	9.07E-05	5.71E-03
Cadmium, urine	GrimAge	2.22	( 1.52, 2.92)	4.43E-04	0.03
Cadmium, urine	GrimAge2	2.38	( 1.53, 3.22)	7.90E-04	0.05

Effect estimates, 95% Confidence Intervals (CI), and unadjusted p-values for each exposure found to be significant after multiple testing adjustment from the no cell-adjustment and cell-adjusted sensitivity models considering serum cotinine levels. Abbreviations: HpCDD: 1,2,3,4,6,7,8-Heptachlororodibenzo-p-dioxin.

**Figure 3:**
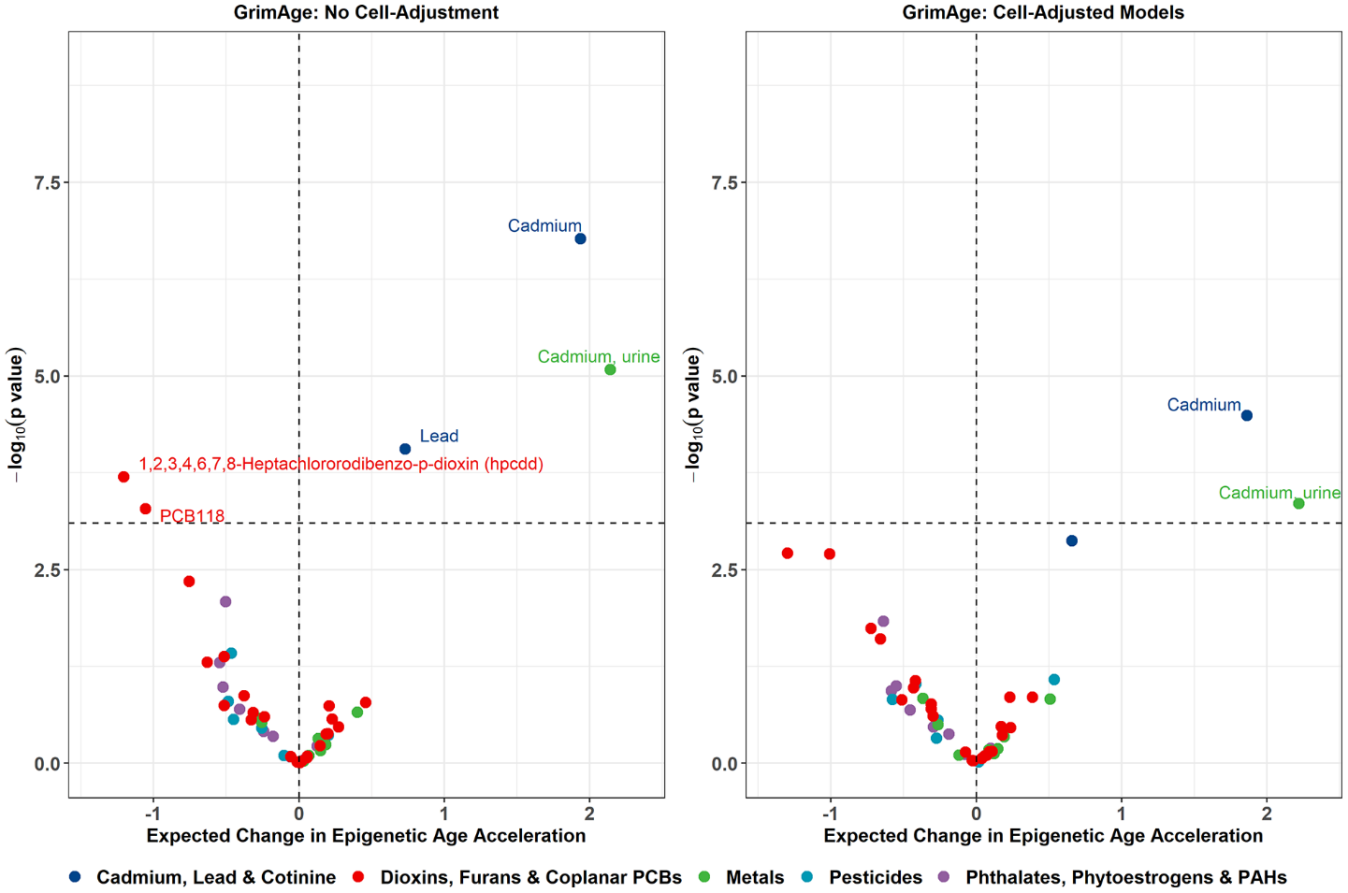
**Volcano plots displaying the expected change in GrimAge acceleration for a 1 SD increase in log_2_-transformed exposure on the X-axis, and -log_10_ p-values on the Y-axis from the sensitivity models adjusting for cotinine exposure.** Color corresponds to the broad category of exposure. Volcano plots for remaining epigenetic clocks are presented in Supplementary Figure 4.

Additionally, several other associations emerged when adjusting for cotinine levels as a proxy for current smoking activity. A 1-SD increase in log_2_ lead exposure was associated with higher GrimAge acceleration (beta = 0.73 years, p = 8.80e-05) and higher GrimAge2 acceleration (beta = 0.67 years, p = 3.90e-04). A 1-SD increase in log_2_ urinary cadmium exposure was associated with higher GrimAge acceleration (beta = 2.14 years, p = 8.34e-06), higher GrimAge2 acceleration (beta = 2.32 years, p = 2.64e-05), and higher DunedinPoAm (beta = 0.03, p = 2.35e-04). 1-SD increase in log_2_ 1,2,3,4,6,7,8-Heptachlororodibenzo-p-dioxin (HpCDD) exposure was associated with lower GrimAge acceleration (beta = -1.21 years, p = 2.01e-04) and lower GrimAge2 acceleration (beta = -1.18 years, p = 6.07e-04). 1-SD increase in log_2_ PCB118 exposure was associated with lower GrimAge acceleration (beta = -1.06 years, p = 5.22e-04) and lower GrimAge2 acceleration (beta = -1.14 years, p = 7.50e-04). Only the associations between serum cadmium and GrimAge, GrimAge2, and DunedinPoAm, as well as the associations between urinary cadmium and GrimAge and GrimAge2, remained significant after cell-type adjustment. Volcano plots visualizing the effect estimates and raw p-values for all exposures from the GrimAge models are shown in Figure 3, with volcano plots for the remaining clocks displayed in Supplementary Figure 4.

Furthermore, we performed a sensitivity analysis by rerunning the primary analysis after imputation of missing covariates. The observed associations closely resembled results from the primary analysis, with consistent positive associations between blood cadmium exposure and GrimAge, GrimAge2, and DunedinPoAm, as well as positive associations of urinary cadmium with GrimAge and GrimAge2 in the non-cell-adjusted models (Supplementary Table 4). No associations remained significant after multiple testing adjustment in the covariate-imputed cell-adjusted models. Volcano plots visualizing the effect estimates and raw p-values for all exposures are displayed in Supplementary Figure 5.

## DISCUSSION

We performed a systematic exposome-wide association study to examine associations between 64 environmental chemical exposures and epigenetic age acceleration in a representative sample of US adults from NHANES. Environmental chemical exposures represent a key modifiable risk factor impacting human health and longevity, and our findings provide evidence for associations between several environmental exposures and epigenetic aging in a large sample representative of the US adult population. Our findings suggest that cadmium is among the strongest environmental chemicals influencing EAA in the general US population, with additional identified associations involving cotinine, lead, PCBs, and dioxins.

We found evidence for EAA in response to exposure to several metals in the NHANES population, including lead and cadmium. Lead exposure has been previously found to be associated with higher GrimAge acceleration in a sample of 290 adults from the Detroit Neighborhood Health Study [17]. We additionally saw consistent strong associations between blood cadmium levels and EAA across several epigenetic clocks. Urinary cadmium levels also exhibited similar magnitude associations, albeit with wider confidence intervals likely related to the smaller available sample sizes for urinary metal measurements. Cadmium has been previously found to be associated with higher EAA across several clocks including GrimAge and DunedinPACE in 2,301 participants from the Strong Heart Study [18]. Tobacco smoke is a major source of cadmium exposure in the general population, [19] and smoking has been found to be strongly associated with both altered DNA methylation [20] and epigenetic aging [21]. This is further supported by our findings of associations between cotinine measurements, a primary metabolite of nicotine and biomarker of tobacco smoke exposure, and increased EAA across several clocks. It is important to note that predicted smoking activity is a direct component of the GrimAge and GrimAge2 predictions, however cotinine was additionally found to be associated with higher DunedinPoAm as well. Our findings of EAA in response to cadmium exposure in primary models adjusted for self-reported smoking activity, in sensitivity models adjusted for cotinine levels, and in smoking status-stratified models suggest that cigarette smoke exposure is not entirely driving associations between cadmium exposure and EAA. Beyond tobacco smoke, diet is a major source of cadmium exposure in the US, providing a pivotal intervention point [22]. Taken together, these findings suggest that cadmium exposure and cotinine levels may be some of the strongest modifiable environmental influences on EAA in the general US population.

We observed negative associations of PCB118 and 1,2,3,4,6,7,8-Heptachlororodibenzo-p-dioxin (HpCDD) exposure with GrimAge/GrimAge2 acceleration in the cotinine-adjusted sensitivity models. PCB118 is a polychlorinated biphenyl and persistent organic pollutant that has been found to be associated with inflammation and hypertension [23, 24]. Interestingly, a suggestive negative association between a related PCB, PCB138, and EAA in children has been previously reported, although this association was found to be attenuated after adjusting for BMI [14]. HpCDD is a Polychlorinated Dibenzo-p-Dioxin, a subclass of dioxins, suggested to act through Aryl Hydrocarbon Receptor (AhR) induction [25]. Little research has examined potential associations between dioxins and epigenetic aging, however, a previous study found positive associations between serum dioxin exposure and sperm epigenetic age among veterans exposed to Agent Orange [26]. Although negative EAA is often considered beneficial or protective, previous research has uncovered seemingly paradoxical relationships of negative EAA among individuals at high risk for colorectal cancer [27]. Interestingly, exposure to persistent organic pollutants, including PCB118 and HpCDD, has been found to be associated with longer telomere length among adult participants in the 2001-2002 NHANES sample, with AhR-mediated telomerase activation suggested as a possible mechanistic explanation [28]. Further research is needed to elucidate the epigenetic mechanisms underlying these observed associations.

We used a stringent multiple testing adjustment to judge statistical significance in our analysis; however, we additionally observed several associations with unadjusted p-value < 0.05 that we highlight in supplemental tables to help inform future research on environmental exposures and epigenetic aging. Among these suggestive associations, Mono-benzyl phthalate (MBzP) was associated with higher Hannum and Skin&Blood age acceleration. Phthalate exposure has been found to be associated with altered EAA in wide range of contexts including in a healthy elderly population, [29] within sperm tissue, [30] and in newborns and children [31, 32]. Other suggestive associations included associations between equol, an isoflavandiol phytoestrogen, and lower GrimAge/GrimAge2 acceleration. Equol is a metabolite of the soy isoflavone daidzein that is produced by gut bacteria in portions of the population [33]. Interestingly, o-Desmethylangolensin (O-DMA), another metabolite of daidzein, was suggestively associated with lower DunedinPoAm and lower GrimAge2 acceleration in the study population as well. Previous research has highlighted associations between individual dietary patterns and epigenetic aging [34]. Future research could seek to clarify whether the decreased EAA in relation to equol and O-DMA exposure observed here is directly related to these compounds, or whether these exposures are indicative of dietary patterns that are driving the associations with EAA.

Our findings are subject to some limitations. Firstly, our analysis was limited to 64 environmental exposures, a small fraction of the true exposome. However, this work substantially expands on previous research with the characterization of associations between several environmental exposures that have not yet been examined in epigenetic aging research. Second, we were only able to examine cross-sectional associations between environmental exposures and EAA in a population of US adults aged 50 years and older. Future research can work to expand on our findings by examining younger populations and early life exposures to identify key periods of susceptibility, using longitudinal samples to examine long term impacts of environmental exposures on EAA, and extending this work to other regions around the world to determine the consistency of the environmental determinants of epigenetic aging globally. Relatedly, our study utilized participants from the 1999-2000 and 2001-2002 NHANES survey cycles, which might not necessarily reflect current exposure patterns or population demographics. Therefore, further research on how the environmental determinants of epigenetic aging may have changed over time is warranted. Third, although we controlled for key confounders including several socioeconomic and health-related variables, there is still potential for residual confounding by factors including dietary patterns and geographical location within the US. Future research can make use of the extensive collection of survey- and laboratory-derived measures available for the NHANES population to investigate the contribution of various other factors in shaping epigenetic aging. Fourth, our analysis only considered EAA measured in blood, inhibiting our ability identify potential tissue-specific associations. Finally, the available sample size varied widely between different exposures. While most analyses were adequately powered, this small sample limitation was especially apparent in the analyses of volatile organic compound exposure for which complete case sample sizes ranged from 96 to 120. The strongest associations were also observed with serum cadmium and cotinine, which also featured the largest sample sizes ranging from 1,856 to 1,893 in the current study sample.

Our work also features some key strengths. By harnessing NHANES data, we were able to conduct an extensive examination of associations between environmental exposures and EAA in a sample that is representative of the US adults ≥50 years. Furthermore, we focused our analysis on exposures measured in biological mediums, providing direct and personal measures of exposure for each chemical. Finally, NHANES features an extensive array of publicly available questionnaire and laboratory data within the same study population, allowing researchers to systematically examine the determinants and consequences of epigenetic aging in a large US population.

Environmental chemical exposures are a key modifiable risk factor and target for interventions seeking to improve human health and longevity. Our study systematically examined associations between 64 common environmental chemical exposures and epigenetic age acceleration among a representative sample of U.S. adults from NHANES, providing a significant advancement in our understanding of the contributions of environmental chemical exposures towards biological aging in the general population. Our findings both reinforce previous findings related to cadmium and cotinine exposure, while also revealing novel associations related to exposures including dioxins and PCBs.

## MATERIALS AND METHODS

### Study population

Our analysis harnessed data from the 1999-2000 and 2001-2002 cycles of NHANES. NHANES is a biannual program organized by the National Center for Health Statistics (NCHS) to collect data from a sample which is representative of the non-institutionalized US population [16]. NHANES datasets consist of a wide range of measures including demographics, physical examination data, and laboratory measurements. This specific subsample included 2,532 adult participants aged ≥ 50 years surveyed in 1999-2000 or 2001-2002 that had blood samples available for DNA methylation analysis. For protection of participant privacy, all NHANES participants aged 85 and above in these cycles were top-coded as 85 years of age. Due to inability to determine true age in participants labelled as 85 years of age and the potential for subsequent systematic bias in the calculation of EAA, all participants ≥ 85 years of age (N = 130) were removed. Additionally, N = 56 participants whose DNAm-derived predicted sex did not match their reported sex were removed, leaving N = 2,346 participants available for analysis. All NHANES participants provided written informed consent and study protocols were approved by the NCHS Research Ethics Review Board.

### Environmental exposures

Laboratory measurement datasets from both waves were acquired from the NHANES website, including 1) Phthalates, Phytoestrogens & Polycyclic aromatic hydrocarbons (PAHs) - Urine, 2) Pesticides - Current Use - Urine, 3) Metals - Urine, 4) Dioxins, Furans, & Coplanar PCBs, 5) Cadmium, Lead, Mercury, Cotinine & Nutritional Biochemistries, and 6) Volatile Organic Compounds (VOCs) - Blood & Water. We did not consider environmental exposures measured in only one of the two waves, measured in pooled samples, or measured in non-biological mediums (*e.g*. dust and water). For Dioxins, Furans, and Coplanar PCBs, we focused analysis only on lipid-adjusted measures. We excluded nutrition-related compounds from the Cadmium, Lead, Mercury, Cotinine & Nutritional Biochemistries dataset, as well as mercury measures which were only characterized in small subsets of the population. We further excluded all measures with detection frequencies below 50% in the study population, calculated using the accompanying compound detection codes or compiled data accessed from Kaggle [35]. This left a total of 64 exposures, of the possible 111 exposures after inclusion criteria were met, available for analysis. All exposure measures were log_2_ transformed to normalize exposure distributions, then centered and scaled to aid in interpretation of model coefficients. Sample sizes for complete case analysis, detection frequencies, and the survey-weighted 0^th^, 25^th^, 50^th^, 75^th^, and 100^th^ percentiles of each exposure measure are presented in Supplementary Table 1. Pearson correlation coefficients between all log_2_-transformed exposure measures are presented in Supplementary Figure 2, however, we note that not all exposures were measured in overlapping subsets of the overall population.

### DNA methylation

Epigenetic age estimates and DNAm-derived cell proportion estimates were downloaded from the NHANES website, and detailed methodology for DNA methylation analysis and processing is provided on the NHANES website [36]. Briefly, DNA was extracted from whole blood from a selection of NHANES adult participants aged ≥ 50 years surveyed in 1999-2000 or 2001-2002. DNA methylation was measured with the Illumina EPIC BeadChip array. We focused analysis on the Horvath panTissue, Hannum, Skin&Blood, PhenoAge, GrimAge, GrimAge2, DunedinPoAm, and DNAmTL clocks [3–10]. Corresponding EAA measures were calculated for each of the epigenetic clocks, except for DunedinPoAm, by extracting the residuals from a regression of chronological age in years on epigenetic age. DunedinPoAm was left untransformed for analyses. Pearson correlation coefficients and median absolute error (MAE) were used to assess the fit between each epigenetic clock whose units are expressed in years and chronological age. Pearson correlation coefficients were also used to assess the correlation between each EAA measure, as well as the DunedinPoAm measure (Supplementary Figure 1).

### Statistical analysis

Potential confounders and precision variables were identified *a priori* and included chronological age in years (continuous), chronological age squared, sex (male vs. female), self-identified race/ethnicity (Non-Hispanic White, Mexican American, Other Hispanic, Non-Hispanic Black, Other Race - Including Multi-Racial), body mass index (BMI) (continuous), poverty to income ratio (PIR), smoking status (Never, Former, Current), education (less than high school, high school diploma or GED, greater than high school education), and occupation category. Never smoking status was defined as not having smoked at least 100 cigarettes in life, former smoking was defined as having smoked at least 100 cigarettes in life but reporting not currently smoking cigarettes, and current smoking was defined as having smoked at least 100 cigarettes in life and reporting currently smoking every day or some days. Occupation was categorized into five groups corresponding to white-collar and professional work, white-collar and semi-routine work, blue-collar and high-skill work, blue-collar and semi-routine work, or no work as previously described [37]. Urinary creatinine was additionally included as a covariate for all exposures measured in urine. To investigate the potential role of cell proportions, we additionally ran models adjusted for the same covariate set plus DNAm-derived cell proportion estimates (CD8, CD4, Nkcells, Bcell, Monocytes, and Neutrophils) [38–40]. Survey-design weighted generalized linear regression models were conducted using the R *Survey* package to account for participant sample weights and the NHANES survey design, using the weights provided with the epigenetic clock dataset [41]. Associations between each log_2_-transformed, centered, and scaled exposure measure and each EAA measure were assessed using the svyglm R function with the covariate sets defined above. Default analyses were all conducted using complete case analysis. Specifically, 84 participants were missing BMI, 267 were missing poverty to income ratio, 2 were missing education category, 137 were missing occupation category, and 6 were missing smoking activity, leaving an effective max sample size of N = 1,895 for analysis.

Serum cotinine, the primary metabolite of nicotine, was included as an environmental exposure in the primary analysis. To better determine the extent to which associations might be driven by current smoking activity, we performed a sensitivity analysis by rerunning the primary models while adjusting for serum cotinine levels in place of self-reported smoking activity. We then performed an additional sensitivity analysis imputing missing covariates using multiple imputation by chained equations with the MICE function with a setting of 5 iterations, repeating the primary analyses described above, and pooling estimates from each imputed dataset using the pool function in R [42]. Associations were considered significant if they exhibited a Bonferroni-adjusted p-value<0.05, with the number of independent tests set to the number of exposures (64 for the main analyses or 63 for the cotinine sensitivity analyses). All analyses were performed in R version 4.2.3. All data used for this analysis is publicly available from the NHANES website (https://www.cdc.gov/nchs/nhanes/index.htm). All code necessary to reproduce this analysis is available on GitHub (https://github.com/D-Khodasevich/NHANES_ExWAS).

## Supplementary Material

Supplementary Figures

Supplementary Table 1

Supplementary Table 2

Supplementary Table 3

Supplementary Table 4
