# Supplementary material for: Exposome-wide association study of environmental chemical exposures and epigenetic aging in the national health and nutrition examination survey
Source: Aging (Albany NY). 2025 Feb 11;17(2):408–30. doi: 10.18632/aging.206201 (PMC11892924; doi:10.18632/aging.206201)
Supplement: Supplementary Figures [file aging-17-206201-s001.pdf]

SUPPLEMENTARY FIGURES

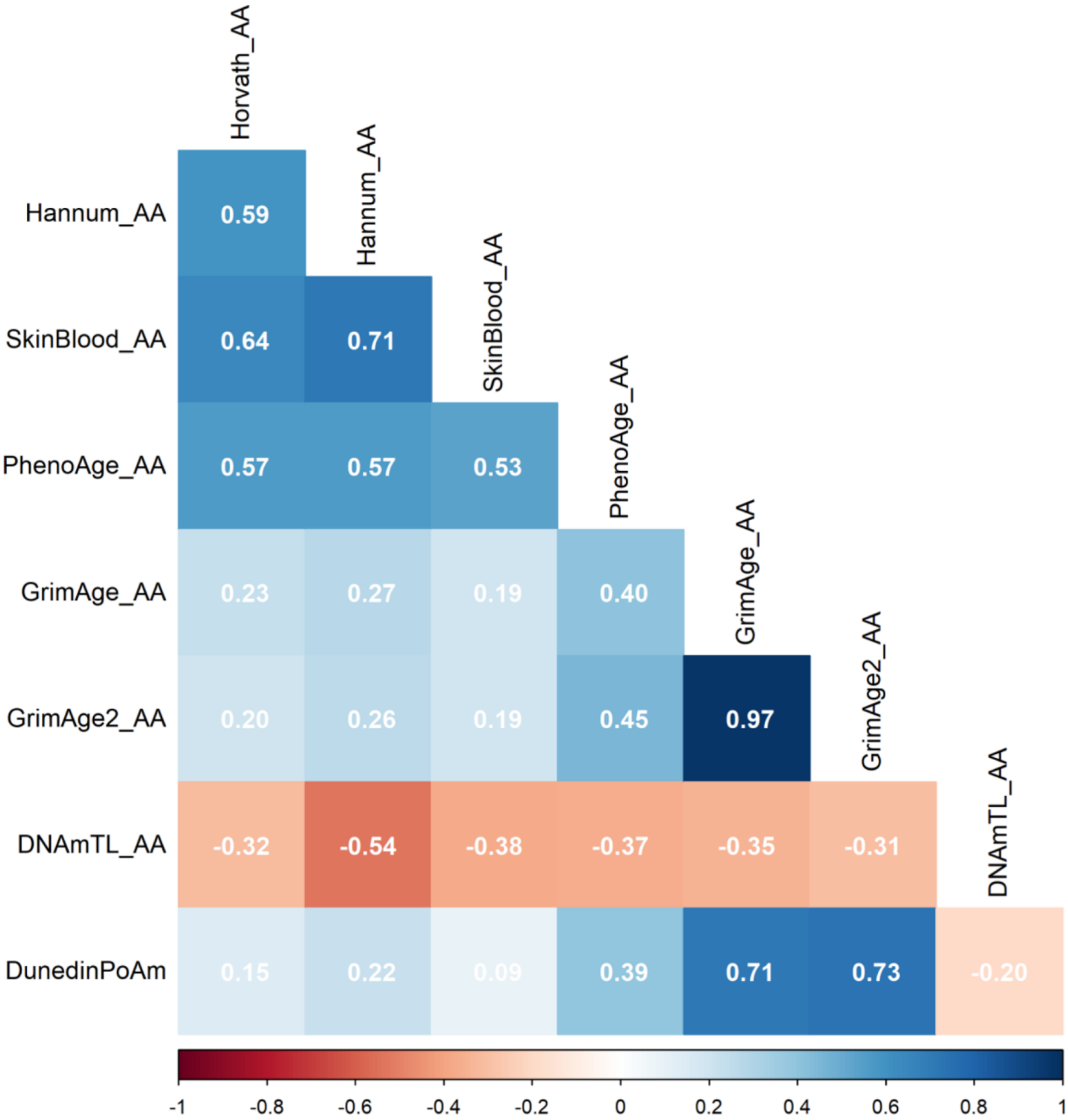

Supplementary Figure 1. Pearson correlation coefficients between epigenetic age acceleration measures from each epigenetic clock for the study sample (N = 2,346).

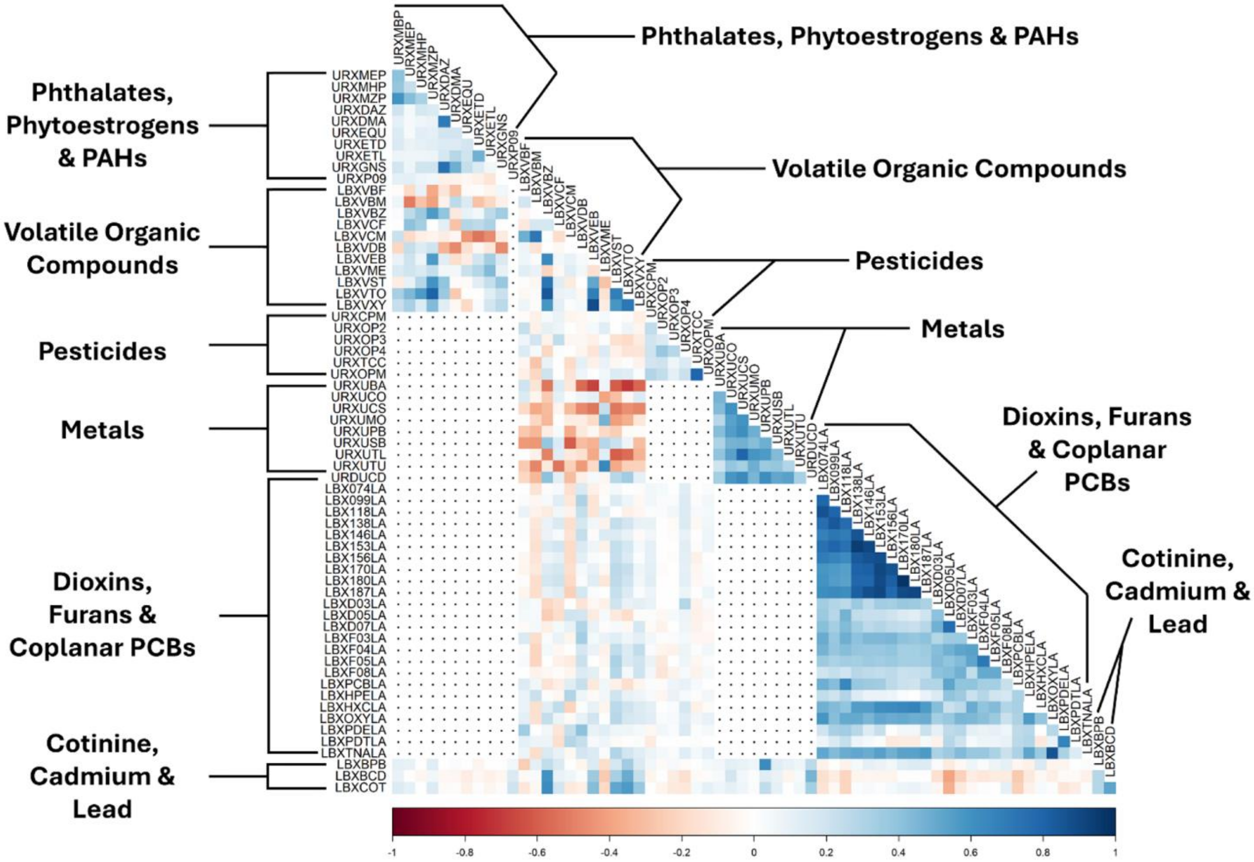

Supplementary Figure 2. Pearson correlation coefficients between each log$_2$-transformed environmental exposure. Black points represent exposure comparisons with 0 overlapping study subjects.

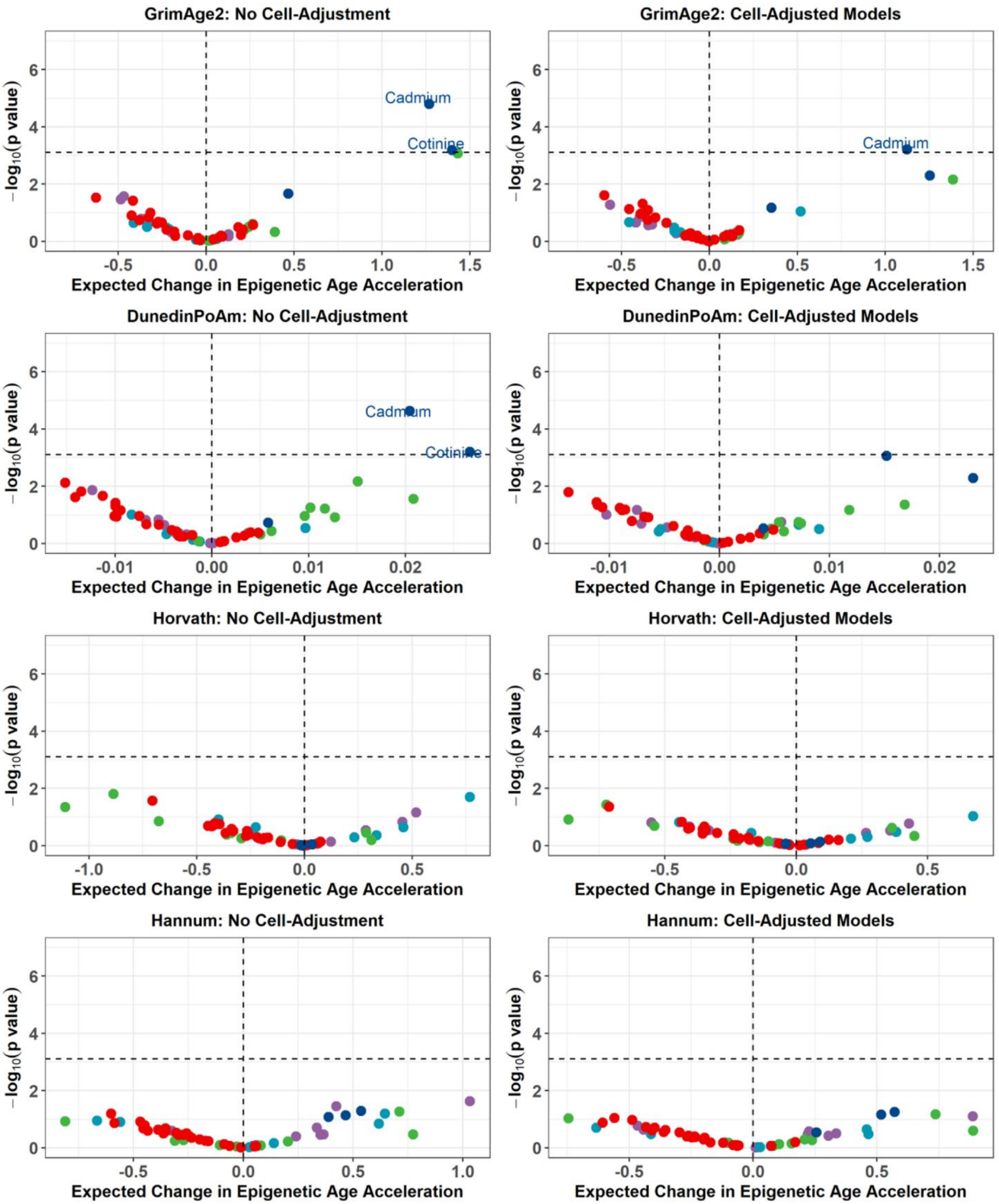
GrimAge2: No Cell-Adjustment
GrimAge2: Cell-Adjusted Models
DunedinPoAm: No Cell-Adjustment
DunedinPoAm: Cell-Adjusted Models
Horvath: No Cell-Adjustment
Horvath: Cell-Adjusted Models
Hannum: No Cell-Adjustment
Hannum: Cell-Adjusted Models
Cadmium
Cotinine
−log10(p value)
Expected Change in Epigenetic Age Acceleration

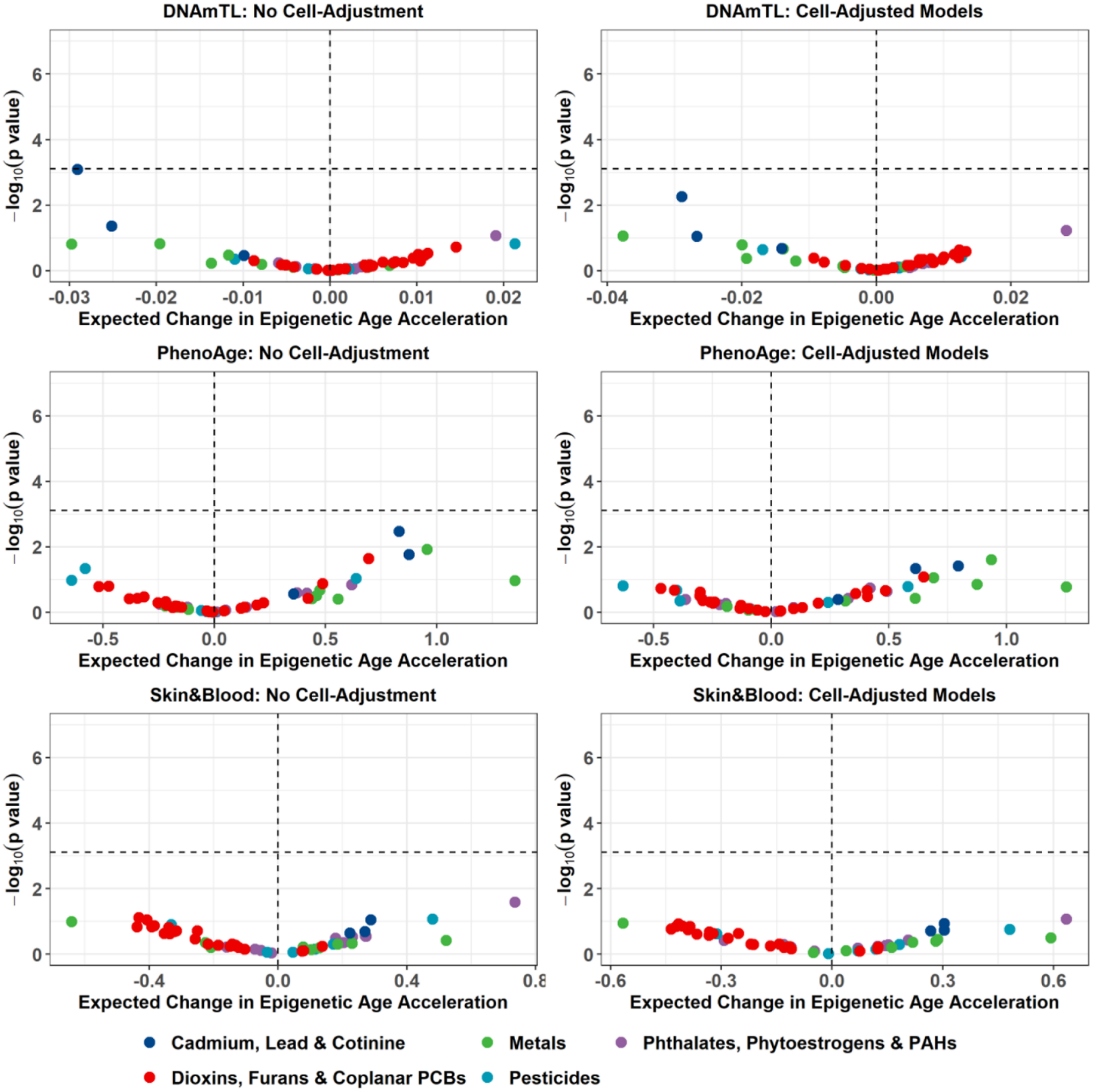

Supplementary Figure 3. Volcano plots displaying the expected change in epigenetic acceleration for a 1 SD increase in $\log_2$-transformed exposure on the X-axis, and $-\log_{10}$ p-values on the Y-axis from the primary models. Color corresponds to the broad category of exposure. Volcano plots for GrimAge are presented in the main text.

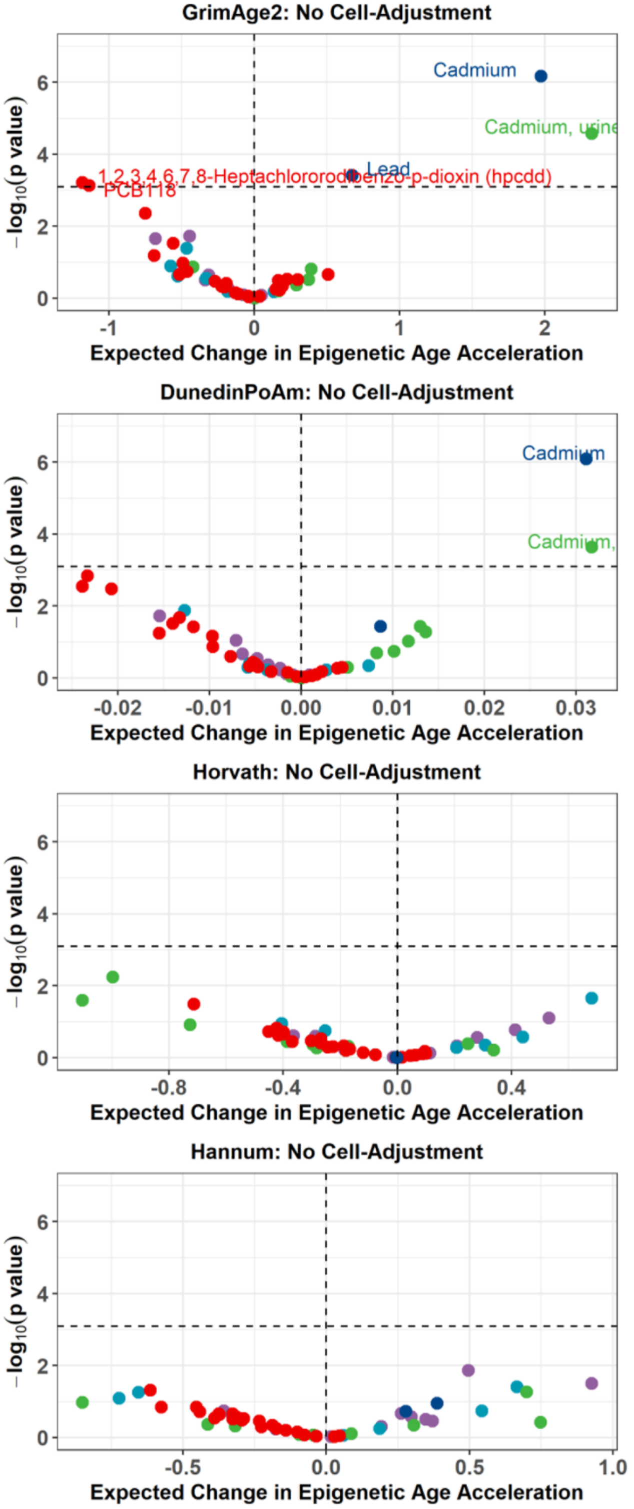
GrimAge2: No Cell-Adjustment
Cadmium
Cadmium, urine
1,2,3,4,6,7,8-Heptachlorodibenzo-p-dioxin (hpcdd)
Lead
PCB118
GrimAge2: Cell-Adjusted Models
Cadmium
Cadmium, urine
DunedinPoAm: No Cell-Adjustment
Cadmium
DunedinPoAm: Cell-Adjusted Models
Cadmium
Horvath: No Cell-Adjustment
Horvath: Cell-Adjusted Models
Hannum: No Cell-Adjustment
Hannum: Cell-Adjusted Models
−log10(p value)
Expected Change in Epigenetic Age Acceleration

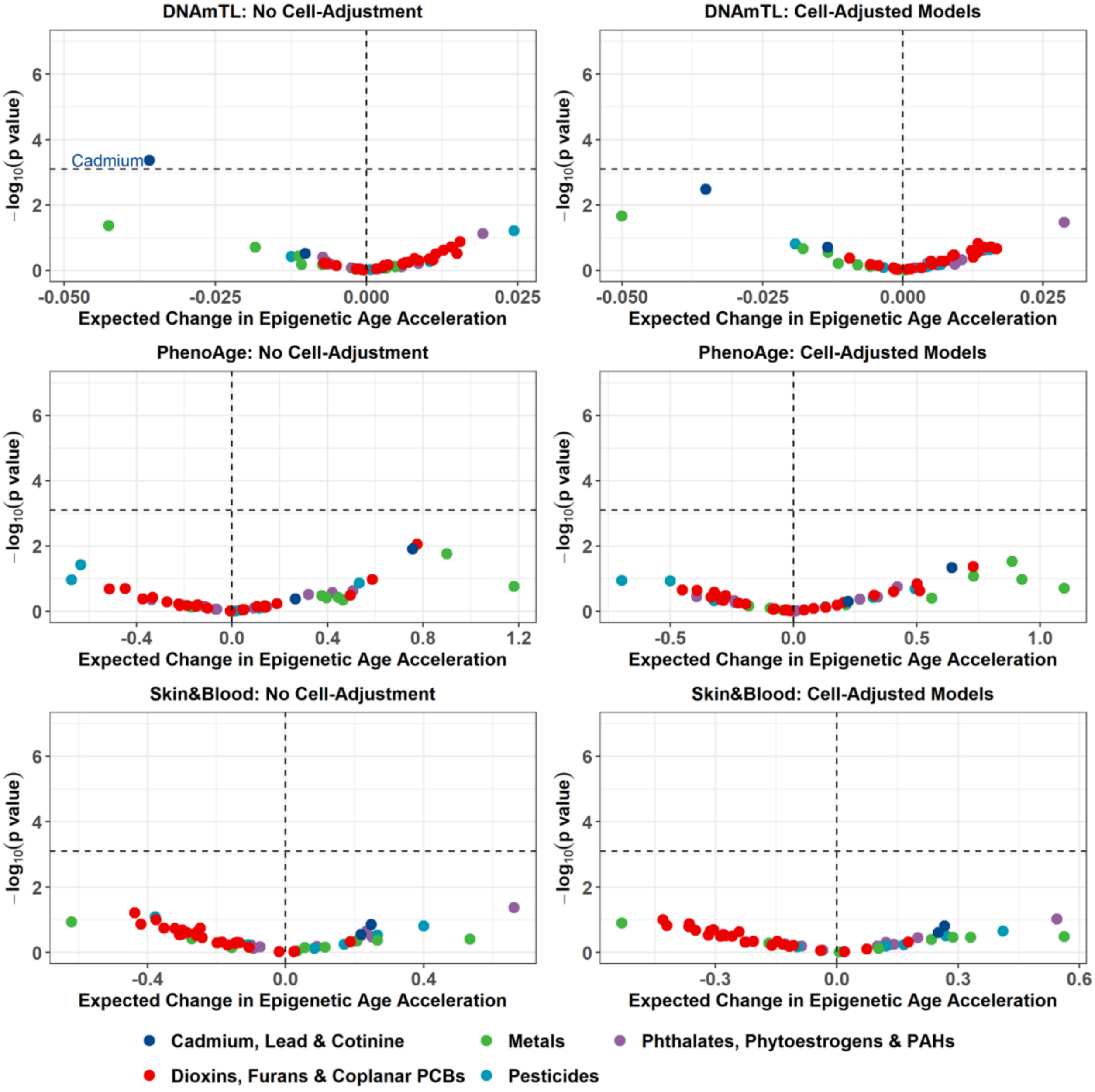

Supplementary Figure 4. Volcano plots displaying the expected change in epigenetic acceleration for a 1 SD increase in log_2-transformed exposure on the X-axis, and $-log_{10}$ p-values on the Y-axis from the sensitivity models adjusting for cotinine exposure. Color corresponds to the broad category of exposure. Volcano plots for GrimAge are presented in the main text.

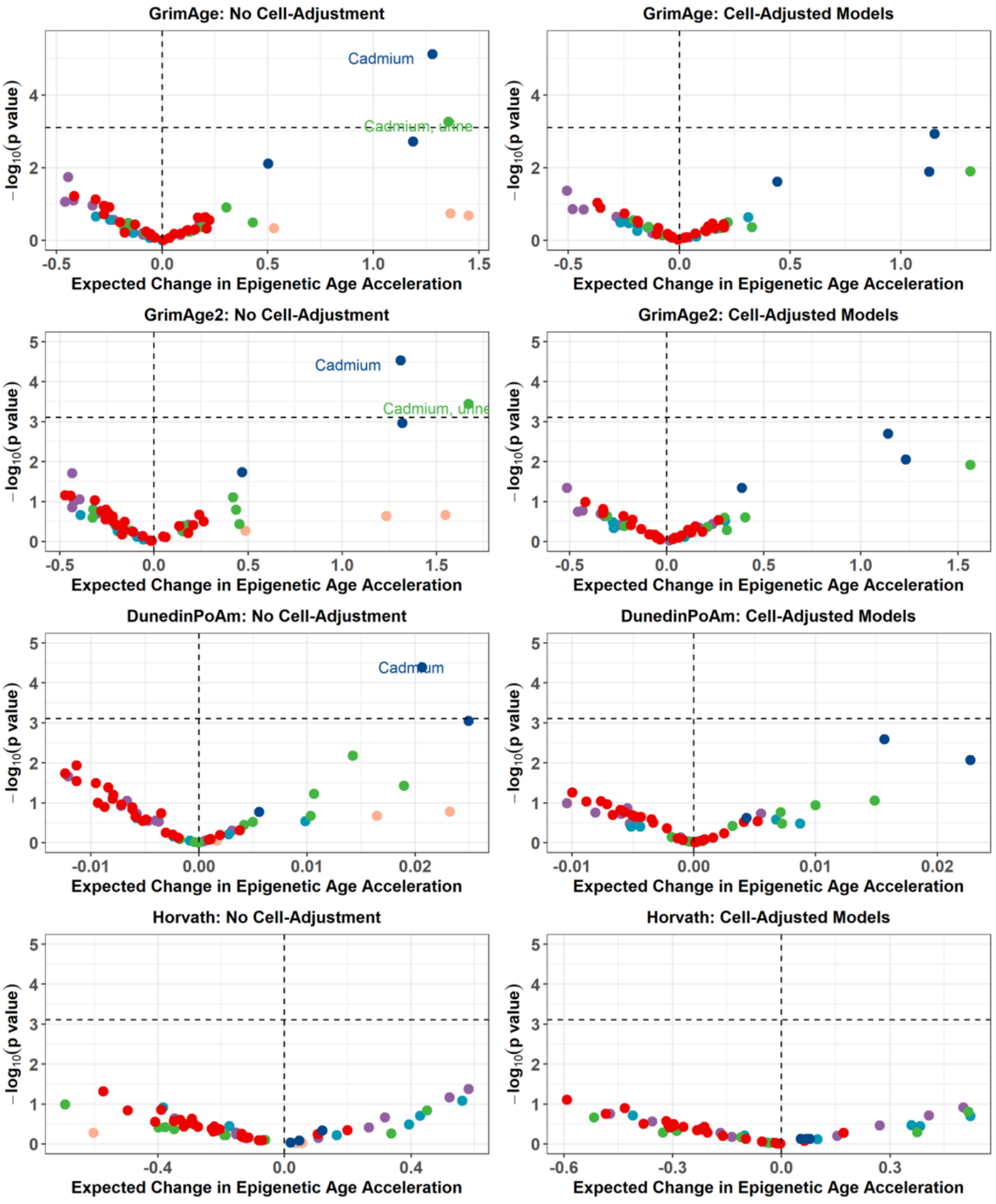

GrimAge: No Cell-Adjustment
GrimAge: Cell-Adjusted Models
GrimAge2: No Cell-Adjustment
GrimAge2: Cell-Adjusted Models
DunedinPoAm: No Cell-Adjustment
DunedinPoAm: Cell-Adjusted Models
Horvath: No Cell-Adjustment
Horvath: Cell-Adjusted Models
Cadmium
Cadmium, urine
$-\log_{10}$(p value)
Expected Change in Epigenetic Age Acceleration

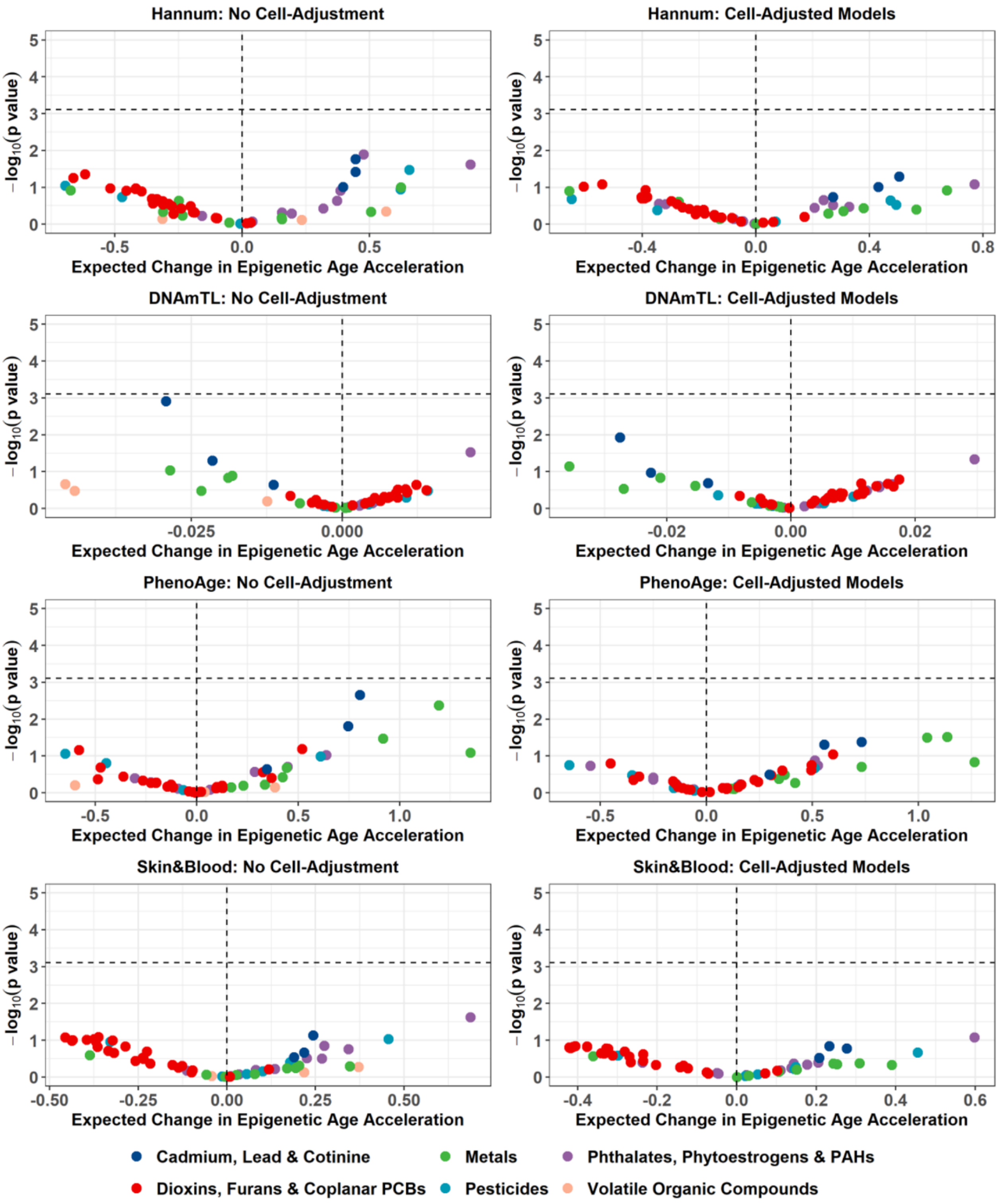

Supplementary Figure 5. Volcano plots displaying the expected change in epigenetic acceleration for a 1 SD increase in log_2-transformed exposure on the X-axis, and $-log_{10}$ p-values on the Y-axis from the sensitivity models considering imputation of missing covariates. Color corresponds to the broad category of exposure.
